# Dynamic change of novel systemic inflammation markers in predicting perinatal outcomes after antenatal corticosteroids in preeclampsia

**DOI:** 10.1097/MD.0000000000046370

**Published:** 2025-12-12

**Authors:** Şebnem Karagün, Ahmet Zeki Nessar, Yusuf Dal, Sefanur Gamze Karaca, Mürşide Çevikoğlu Killi, Hamza Yildiz, Ayhan Coşkun

**Affiliations:** a Department of Obstetrics and Gynecology, Division of Perinatology, Mersin University Faculty of Medicine, Mersin, Turkey; b Department of Obstetrics and Gynecology, Division of Gynecological Oncology Surgery, Mersin University Faculty of Medicine, Mersin, Turkey; c Department of Obstetrics and Gynecology, Mersin University Faculty of Medicine, Mersin, Turkey.

**Keywords:** antenatal corticosteroids, inflammatory markers, perinatal outcomes, preeclampsia, ΔAPRI, ΔMPV

## Abstract

Preeclampsia (PE) is characterized by systemic inflammation and endothelial dysfunction, contributing to adverse maternal and neonatal outcomes. Antenatal corticosteroids (ACS) therapy is widely used to enhance fetal lung maturity in women at risk of preterm birth. However, little is known about the dynamic changes in maternal inflammatory markers after ACS administration in PE and their potential predictive value for perinatal outcomes. This retrospective cohort study included 206 pregnant women with PE who received ACS therapy due to preterm birth risk. Complete blood count (CBC) parameters and systemic inflammatory indices were recorded before and after ACS. Delta (Δ) values were calculated to reflect the percentage change between pre- and post-ACS measurements. Correlations between Δ inflammatory markers and perinatal outcomes were analyzed, and predictive performance was assessed using receiver operating characteristic (ROC) curve analysis. Following ACS administration, neutrophil-to-lymphocyte ratio (NLR), systemic immune-inflammation index (SII), and aspartate aminotransferase-to-platelet ratio (APRI) increased significantly, while lymphocyte-to-monocyte ratio (LMR) and platelet-to-lymphocyte ratio (PLR) decreased. Δ mean platelet volume (MPV), Δ aspartate aminotransferase (AST), Δ Lactate Dehydrogenase (LDH), ΔNLR, and ΔAPRI showed low-level positive correlations with umbilical cord blood pH. ROC analysis indicated that ΔMPV ≤ 1.81 (area under the curve (AUC) = 0.664) and ΔAPRI ≤ 1.81 (AUC = 0.605) moderately predicted neonatal intensive care unit (NICU) admission. Dynamic changes in maternal inflammatory markers occur after ACS therapy in preeclamptic pregnancies. ΔMPV and ΔAPRI demonstrated potential predictive value for NICU admission, highlighting their possible clinical utility in risk stratification. These findings provide novel insight into the maternal inflammatory response to ACS in PE and its implications for perinatal outcomes.

## 1. Introduction

Preeclampsia (PE) is a significant global public health concern, affecting 2 to 8% of all pregnancies.^[1]^ The reported prevalence of PE in Turkey is comparable to global estimates, with an incidence of approximately 3.2%.^[2]^ Annually, more than 500,000 neonatal deaths and over 70,000 maternal fatalities are attributed to PE, impacting approximately 4 million women.^[3]^ Although the maternal mortality rate in PE has decreased, prematurity has devastating effects in the long term.^[4]^ The management of PE requires a multidisciplinary approach aimed at controlling severe hypertension, preventing seizures, and optimizing both maternal and fetal outcomes through vigilant monitoring.^[5,6]^ The timing of delivery remains the cornerstone of management and is determined by gestational age, disease severity, and maternal–fetal condition.^[7,8]^ Ultimately, delivery is the only definitive treatment, as it halts disease progression and prevents potentially life-threatening complications such as eclampsia and HELLP syndrome.^[6,9]^ A quarter of pregnant women diagnosed with PE are susceptible to premature delivery.^[10]^ In both full-term and preterm infants, PE increases the likelihood of neonatal respiratory distress syndrome.^[11]^ Antenatal corticosteroid (ACS) therapy reduced perinatal death due to preterm birth by reducing the frequency and severity of respiratory comorbidities.^[12]^

It is an acknowledged fact that immune dysregulation, which is involved in the pathogenesis of PE, is causative of an excessive and prolonged inflammatory response.^[13]^ Systemic inflammation activated by endothelial dysfunction in PE contributes to placental ischemia.^[14]^ Consequently, readily available systemic inflammatory markers derived from routine complete blood count (CBC) parameters – such as the neutrophil-to-lymphocyte ratio (NLR), systemic immune-inflammation index (SII), have attracted growing interest as potential indicators of disease severity and predictors in PE.^[15,16]^ The current focus is on the prediction of maternal and neonatal outcomes by CBC inflammatory markers involved in pathogenesis.^[17,18]^ A meta-analysis by Kang et al demonstrated that NLR serves as a significant marker for predicting PE severity, while Aslan and colleagues further reported its specific association with fetal loss in cases of severe PE, underscoring its prognostic value in adverse perinatal outcomes.^[19,20]^

Nayeri et al conducted a study that examined alterations in inflammatory markers prior to and following ACS therapy in PE.^[21]^ Significant reductions were observed in the levels of C-reactive protein (CRP) and interleukin-6 (IL-6). A study on inflammation in premature neonates demonstrated that administration of ACS treatment resulted in a decrease in neutrophil count.^[22]^ The change in NLR according to trimesters was shown to be predictive of PE in the study of Bulbul et al.^[23]^ Nevertheless, there exists a dearth of evidence concerning the alteration in maternal blood inflammatory parameters induced by ACS treatment in PE.

However, the extent to which ACS therapy modulates the maternal inflammatory response in PE and its implications for perinatal outcomes remains unclear. To address this knowledge gap, this study therefore aimed to evaluate the predictive significance of dynamic changes in maternal systemic inflammatory markers, derived from CBC parameters, before and after ACS administration in preeclamptic pregnancies at risk of preterm birth.

## 2. Materials and methods

In this retrospective cohort study, a total of 206 pregnant women who were followed up in the Perinatology Clinic of Mersin University Hospital between January 1, 2020, and October 31, 2023, due to PE and who received ACS treatment due to the risk of preterm delivery were included. The study was approved by the Mersin University Ethics Committee with decision number 2023/775. The Declaration of Helsinki’s principles guided the conduct of this study. Diagnosis of PE, pregnancy follow-up, routine blood tests before and after ACS, and delivery of all patients enrolled in the study were carried out in our hospital. All the data used in the study were obtained anonymously from the hospital’s electronic database. Informed consent could not be obtained due to the anonymous analysis of the data and the retrospective design.

## 3. Study design and population

**Figure 1.**
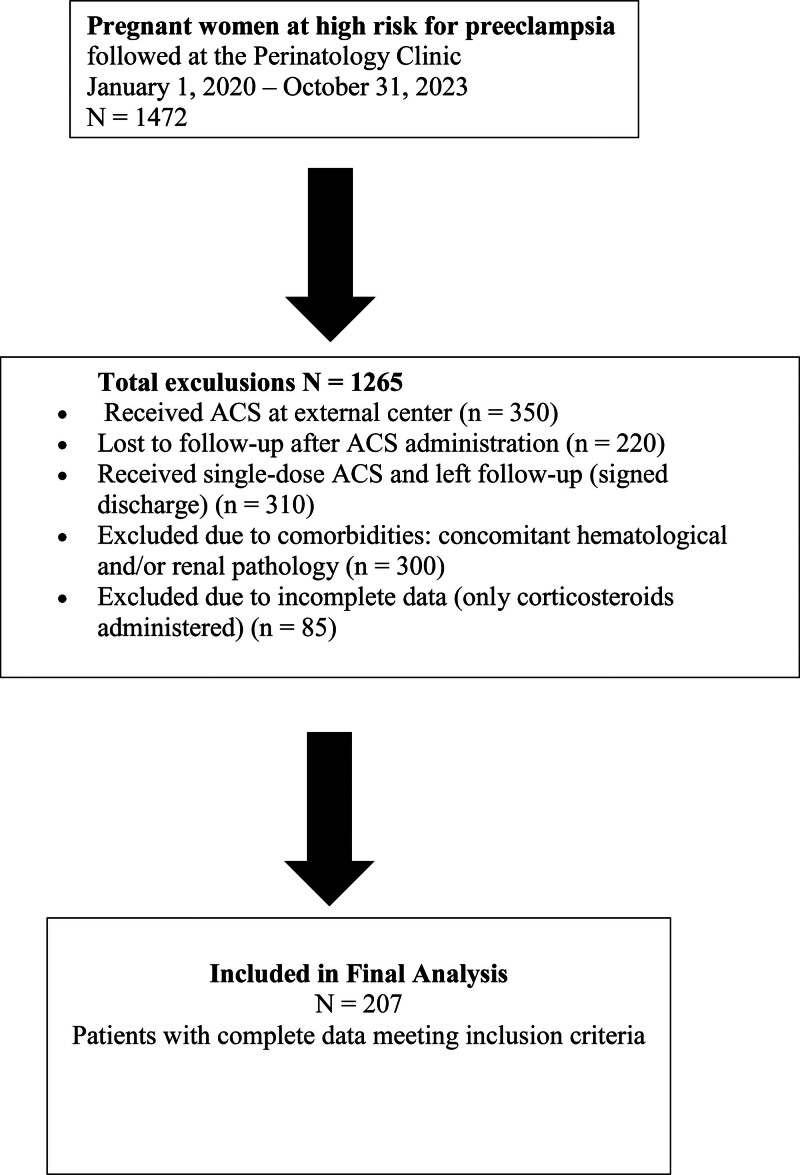
Flowchart of patient selection.

Criteria for the diagnosis of PE were based on ACOG Practice Bulletin, Number 222.^[6]^ Women were considered at risk of preterm labor if they were likely to deliver before 37 weeks’ gestation.^[24]^ The use of ACS in eligible pregnant women at risk of preterm delivery is part of our clinical algorithm. A protocol for fetal lung maturation administered 2 12-mg intramuscular doses of betamethasone 24 hours apart to pregnant women between 24 0/7 weeks and 33 6/7 weeks of gestation who were at risk of preterm birth.^[25]^ The following criteria are to be excluded from the study: multiple pregnancies, known hypertensive status other than PE, known fetal anomalies, maternal autoimmune or chronic inflammatory disease, the presence of active infection and diabetes, and pregnant women receiving a single rescue dose of betamethasone. A participant flow diagram is provided in Figure 1.

## 4. Assessment of CBC parameters and indices

The study employed 2 time periods for the analysis of CBC values. The first period spanned the measurement of CBC values before the commencement of treatment on the day of ACS planning (pre-ACS), while the second period encompassed the measurement of CBC values on the day following the completion of 2 doses of ACS treatment (post-ACS). Venous blood samples were collected from the subjects in 3 mL EDTA tubes (SamplixVR, Greiner Bio One, Austria) and analyzed using an automated hematology analyzer (Beckman Coulter Gen-S system device).

The blood parameters evaluated for the study are: platelet (PLT, ×10^9^/L), red cell distribution (RDW, %), mean corpuscular volume (MCV, fL), mean platelet volume (MPV, fL), neutrophil (N) and lymphocyte (L) count (×10^9^/L), lactate dehydrogenase (LDH U/L), aspartate aminotransferase (AST, U/L), and alanine aminotransferase (U/L). Inflammation scores were calculated as follows: NLR = neutrophil count (NC)/ lymphocyte count (LC); lymphocyte-to-monocyte ratio (LMR) = lymphocyte count/monocyte count; PLR = platelet count (PC)/ LC; aspartate aminotransferase-to-platelet ratio (APRI): Aspartate transaminase (AST)/platelet count; SII = PC × NC/LC; SIRI = MC × NC/LC; derived neutrophil-to-lymphocyte ratio (DNLR) = NC/(WBC − NC). We calculated the change in inflammatory parameters pre-ACS and post-ACS as a percentage and named as the delta (Δ) value.

The medical records of each of the cases that met the inclusion criteria were reviewed and the following variables were recorded in the data set: maternal age, demographic information, body mass index, gestational age at which ACS was administered, gestational age at birth, birth weight, appearance, pulse, grimace, activity, and respiration (APGAR) scores for the first and fifth minutes, admission to neonatal intense care unit, neonatal outcome, and pre- and post-ACS CBC parameters and indices.

## 5. Statistical analyses

The data were analyzed using the SPSS (Statistical Package for Social Sciences) 18.0 software package. In descriptive analyses, frequency data were presented as numbers (n) and percentages (%), while numerical data were presented as arithmetic means ± standard deviations (SD) and medians (1st–3rd quartiles (IQR)). The suitability of the numerical data for normal distribution was examined using the Kolmogorov–Smirnov test. The distribution of numerical variables that did not exhibit a normal distribution in 2 dependent groups was evaluated using the Wilcoxon signed-rank test. The relationship between non-normally distributed numerical data was examined with Spearman correlation analysis. Correlation relationships were classified as follows: low correlation (*R* = 0.05–0.30), low-moderate correlation (*R* = 0.30–0.40), moderate correlation (*R* = 0.40–0.60), good correlation (*R* = 0.60–0.70), very good correlation (*R* = 0.70–0.75), and excellent correlation (*R* = 0.75–1.00). Diagnostic accuracy of novel blood inflammation markers for fetal outcomes was expressed as the area under the receiver operating characteristic (ROC) curve (area under the curve (AUC)) along with the 95% confidence interval (CI). The cutoff value in the ROC curve analysis was determined based on the point with the highest sum of sensitivity and specificity. The statistical significance level for all tests was set at *P* < .05.

## 6. Results

Two-hundred and 6 preeclamptic pregnant women who received ACS treatment were included in the study. The demographic characteristics of participants and perinatal outcomes are shown in Table 1. The median week of ACS administration was recorded as 31.35 (29.50–33.40) weeks. During the follow-up of the pregnant women, non-reassuring fetal status developed in 58.7% of the pregnant women and the method of delivery was cesarean section in 94.2% of the cases. It was found that 20.9% of the newborns had meconium-stained amniotic fluid and 54.4% of them required neonatal intensive care.

**Table 1:** Demographic characteristics of participants and perinatal outcomes.

Variables (n = 206)	Mean ± SD	Median (IQR)
Maternal age (yr)	30.49 ± 6.58	30.00 (25.00–36.00)
BMI (kg/m^2^)	26.26 ± 4.18	26.10 (23.30–28.92)
Gravida	2.80 ± 1.99	2.00 (1.00–4.00)
Parity	1.27 ± 1.54	1.00 (.00–2.00)
Timing of ACS (wk)	31.20 ± 2.42	31.35 (29.50–33.40)
Birth week (wk)	35.23 ± 3.40	36.15 (33.00–38.00)
APGAR (1st min)	6.35 ± 2.11	7.00 (5.00–8.00)
APGAR (5th min)	8.12 ± 1.67	9.00 (8.00–9.00)
Birth weight (g)	2486.09 ± 892.69	2560.00 (1833.75-3072.50)
Umbilical artery blood pH	7.29 ± 0.10	7.30 (7.26–7.35)

	n	%
Mode of delivery (cesarean section)	194	94.2
Gender (female)	96	46.6
Non-reassuring fetal status (NRFS)	121	58.7
Meconium-stained amniotic fluid (MSAF)	43	20.9
Neonatal intensive care unit (NICU) admission	112	54.4

ACS = antenatal corticosteroids, APGAR = appearance, pulse, grimace, activity, and respiration, BMI = body mass index, IQR = 1st–3rd quartiles.

The distribution of laboratory parameters measured pre-ACS and post-ACS is examined in Table 2. Post-ACS, platelet, LMR and PLR levels decreased significantly; It was determined that the levels of leukocyte, monocyte, neutrophil, RDW-CV, aspartate aminotransferase (AST), NLR, SIRI, APRI, RPR and DNLR increased significantly (P values; *P* < .001; *P* < .001; *P* < .001; *P* = .004; *P* = .003; *P* = .001; *P* = .010; *P* = .001; and *P* = .007). There was no statistically significant difference in the distribution of other laboratory parameters pre-ACS and post-ACS (*P* > .05).

**Table 2:** Maternal blood parameters and indices before (pre-ACS) and after ACS (post-ACS) exposure.

Variables (n = 206)	pre-ACS	post-ACS	*P* value*^,^^†^
MCV (%)	84.00 (80.00–88.00)	84.00 (79.00–87.00)	.645
Leukocyte (10^3^/µL)	10.29 (8.82–12.66)	11.53 (9.25–14.17)	.004
Lymphocyte (10^3^/µL)	1.85 (1.40–2.19)	1.77 (1.36–2.15)	.292
Monocyte (10^3^/µL)	.62 (.46–0.79)	.65 (.50–.84)	.011
Neutrophil (10^3^/µL)	7.62 (6.11–9.74)	8.58 (6.76–11.31)	.001
Platelet (10^3^/µL)	228.00 (177.00–281.25)	206.50 (155.00–257.00)	<.001
PDW (fL)	13.00 (11.00–14.00)	13.00 (11.00–15.00)	.147
RDW-CV (%)	13.80 (13.20–14.70)	14.05 (13.20–15.33)	<.001
MPV (fL)	10.85 (10.07–11.50)	10.95 (10.10–11.63)	.128
ALT (U/L)	12.65 (9.00–19.92)	12.00 (9.00–19.25)	.230
AST (U/L)	19.00 (15.00–29.00)	21.00 (16.00–30.25)	.010
LDH (U/L)	221.00 (169.00–307.75)	230.50 (187.75–287.75)	.424
LMR (%)	2.93 (2.27–4.14)	2.72 (2.08–3.57)	.004
NLR (%)	4.21 (2.96–6.34)	4.89 (3.33–7.35)	.024
PLR (%)	121.45 (92.54–166.71)	110.48 (82.25–160.09)	.003
SII	936.15 (618.73–1428.77)	922.59 (609.82–1554.64)	.853
SIRI	2.48 (1.69–3.80)	3.15 (2.01–5.10)	.001
APRI	0.08 (0.06–0.13)	0.10 (0.07–0.17)	<.001
RPR	0.06 (0.04–0.08)	0.07 (0.06–0.09)	<.001
DNLR	2.85 (2.12–4.14)	3.26 (2.38–4.92)	.007

Median (IQR).

ACS = antenatal corticosteroids, ALT = alanine aminotransferase, AST = aspartate aminotransferase, DNLR = derived neutrophil-to-lymphocyte ratio, LDH = lactate dehydrogenase, LMR = lymphocyte-to-monocyte ratio, MCV = mean corpuscular volume, MPV = mean platelet volume, NLR = neutrophil-to-lymphocyte ratio, RDW = red cell distribution width, RPR = red cell distribution width-to-platelet ratio, SII = systemic immune-inflammation index, SIRI = systemic inflammation response index.

† Wilcoxon signed-rank test.

* *P* < .05.

The change in laboratory parameters and indices post-ACS compared to pre-ACS was calculated as a percentage called delta (Δ), and its relationship with fetal outcomes was evaluated. Table 3 presents the relationship between statistically significant Δ parameters and fetal outcome. There is a negative correlation between birth week and ΔMCV. A positive, low-significant relationship was noted between Δ WBC, Δ neutrophil, ΔMPV, ΔLDH, ΔNLR, Δ SII, and ΔDNLR (r and P values, respectively; *r* = −0.220; *P* = .001; *R* = 0.185; *P* = .008; *P* = .146; *P* = .001; *P* = .160). A low, statistically significant negative correlation was determined between the 1st minute APGAR score and Δ monocytes (*r* = −0.141; *P* = .043). A low, statistically significant negative correlation was determined between the 5th minute APGAR score and ΔMCV (*r* = −0.168; *P* = .016). Birth weight exhibits a negative correlation with ΔMCV and lymphocytes. A low-level positive correlation was detected between ΔMPV, ΔAST, ΔLDH, ΔNLR, and Δ APRI (*r* and *P* values, respectively; *r* = −0.199; *P* = .004; *r* = −0.140; *P* = .045; *R* = 0.168; *P* = .156; *R* = 0.232; *P* = .025; *P* = .014). A positive, low-level, statistically significant correlation was determined between umbilical cord blood pH level and ΔLDH (*r* and *P* values, respectively; *R* = 0.153; *P* = .028; *R* = 0.141; *P* = .043; *R* = 0.156; *P* = .025).

**Table 3:** Correlation of dynamic changes in laboratory parameters and indices with fetal outcome after exposure to ACS.

		Delivery week	APGAR (1st min)	APGAR (5th min)	Birth weight	Umbilical artery blood pH
ΔHgb	*r*	.047	.015	.013	−.019	.153
	*P*	.506	.829	.853	.784	.028
ΔHtc	*r*	.057	.027	.029	−.008	.141
	*P*	.414	.702	.676	.913	.043
ΔMCV	*r*	−.220	−.078	−.168	−.199	−.080
	*P*	.001	.265	.016	.004	.255
ΔWbc	*r*	.185	.029	.055	.096	.001
	*P*	.008	.680	.436	.170	.984
ΔLymphocyte	*r*	−.102	−.105	−.083	−.140	−.030
	*P*	.145	.132	.236	.045	.665
ΔMonocyte	*r*	−.109	−.141	−.117	−.089	−.106
	*P*	.120	.043	.095	.205	.129
ΔNeutrophil	*r*	.204	.055	.084	.115	.020
	*P*	.003	.435	0,228	.099	.780
ΔMPV	*r*	.146	−.032	−.001	.168	−.024
	*P*	.036	.650	.989	.016	.731
ΔAST	*r*	.132	.081	.043	.156	.104
	*P*	.058	.245	.540	.026	.137
ΔLDH	*r*	.249	.002	.024	.232	.156
	*P*	<.001	.981	.730	.001	.025
ΔNLR	*r*	.189	.099	.094	.156	.043
	*P*	.006	.158	.178	.025	.544
ΔSII	*r*	.160	.104	.064	.108	−.028
	*P*	0,021	.138	.359	.122	.688
ΔAPRI	*r*	.118	.071	.060	.170	.110
	*P*	.090	.309	.395	0,014	.115
ΔDNLR	*r*	.168	.110	.109	.131	.046
	*P*	.016	.114	.120	.060	.514

ACS = antenatal corticosteroids, APGAR = appearance, pulse, grimace, activity, and respiration, APRI = aspartate aminotransferase-to-platelet ratio, AST = aspartate aminotransferase, DNLR = derived neutrophil-to-lymphocyte ratio, LDH = lactate dehydrogenase, MCV = mean corpuscular volume, MPV = mean platelet volume, NLR = neutrophil-to-lymphocyte ratio, *r* = Spearman korelasyon katsayisi, SII = systemic immune-inflammation index.

**Figure 2.**
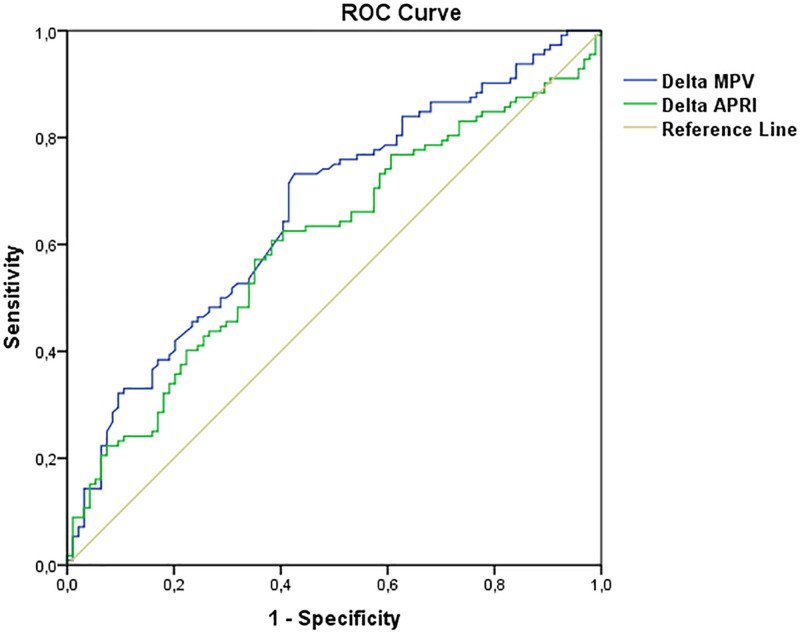
ROC curve for neonatal intensive care unit requirement (NICU) of Delta MPV and Delta APRI parameter. APRI = aspartate aminotransferase-to-platelet ratio, MPV = mean platelet volume, ROC = receiver operating characteristic.

Figure 2 presents the receiver operating characteristic (ROC) curve analysis for ΔMPV and ΔAPRI. For the ΔMPV value, it was determined that values of 1.81 and lower could predict the need for neonatal intensive care unit (NICU) with 71.4% sensitivity and 58.5% specificity (AUC = 0.664 (95% CI = 0.590–0.738; *P* < .001). For the ΔAPRI value, a cutoff point of ≤17.43 predicted the need for NICU with a sensitivity of 62.5% and a specificity of 59.6% (AUC = 0.605; 95% CI = 0.528–0.682; *P* = .010).

## 7. Discussion

### 7.1. Key findings

ACS therapy is well recognized for reducing neonatal respiratory morbidity in preterm birth; however, its effect on maternal inflammatory responses in PE remains uncertain.^[26]^ To our knowledge, this is the first study to investigate the dynamic changes in maternal inflammatory markers following ACS administration in preeclamptic pregnancies and their association with perinatal outcome**s.** Our findings suggest that ΔAPRI and ΔMPV values following ACS administration could serve as potential indicators for NICU admission in preeclamptic pregnancies. Furthermore, we identified significant correlations between specific dynamic parameters and key perinatal indicators. ΔLDH demonstrated a significant positive correlation with both gestational age at delivery and umbilical cord pH, while ΔMCV showed a significant negative correlation with gestational age at delivery. These findings highlight the significant impact of ACS on the maternal inflammatory cascade in PE and establish the potential clinical value of monitoring dynamic changes in readily available hematological parameters for predicting perinatal outcomes.

### 7.2. Clinical relevance of the findings with comparison to previous studies

PE is a multifactorial disorder characterized by a complex pathophysiological cascade involving endothelial dysfunction, intravascular inflammation, and syncytiotrophoblast stress.^[27]^ In recent years, increasing attention has been directed toward evaluating how these inflammatory processes are reflected in maternal CBC parameters.^[28,29]^ However, the impact of ACS therapy on these inflammatory alterations in preeclamptic pregnancies remains less well understood.

In a study by Nayeri et al, the effect of ACS administration on inflammatory processes in PE was examined.^[21]^ The results indicated a transient decrease in IL-6 levels for 48 hours. The increase in inflammatory parameters observed in our study at 24 hours post-ACS may reflect the persistent inflammatory state characteristic of PE, which a single course of corticosteroids may be insufficient to fully suppress. This concept is supported by randomized controlled trials demonstrating that extended steroid administration until delivery improves maternal outcomes in patients with HELLP syndrome – a severe form of PE; characterized by hemolysis, elevated liver enzymes, and low platelet count, suggesting that the inflammatory cascade in severe preeclamptic disorders requires sustained immunomodulation.^[30,31]^ A recent study by Czamara et al demonstrated that ACS is associated with lasting changes in placental DNA methylation (DNAm), promoting a gene expression profile linked to placental dysfunction and increased inflammation.^[32]^ This provides a potential mechanistic basis for our observations: the underlying pro-inflammatory programming in PE placentas may drive a sustained inflammatory response that is not easily reversed by a brief ACS course, manifesting as the increased inflammatory indices we measured in maternal blood.

### 7.3. Correlations with maternal and fetal outcome

In the study by Beser Ozmen, neonatal inflammatory CBC parameters were compared between preterm infants (<34 weeks) exposed and unexposed to ACS.^[22]^ While WBC, CRP, IL-6, and IL-10 levels were similar, the neutrophil count was significantly lower in the full-course ACS group. Likewise, in a larger cohort evaluating the postnatal effects of ACS up to day 28, no significant neonatal anti-inflammatory effect was observed.^[33]^ Similarly, it was observed that the administration of ACS had no effect on postnatal blood inflammatory parameters in newborns born before 32 weeks.^[34]^ Although our study did not directly assess neonatal inflammatory parameters, we identified that post-ACS maternal ΔAPRI and ΔMPV values in preeclamptic pregnancies may serve as potential indicators for predicting the need for NICU admission. This suggests that a blunted increase in these markers following ACS may be a sign of a poorer maternal and fetal adaptive response.

In a prospective study investigating inflammation in threatened preterm labor, NLR, SII, and SIRI values were found to be significantly higher in the preterm group, indicating enhanced systemic inflammation.^[35]^ Similarly, in a large cohort study by Yao et al evaluating PE prediction, elevated neutrophil, lymphocyte, and platelet counts in the second trimester were significantly associated with an increased risk of PE.^[36]^ These findings collectively suggest that both preterm labor and PE involve inflammatory alterations reflected in CBC-derived markers. In our cohort, following ACS administration, platelet, LMR, and PLR levels decreased significantly, while leukocyte, monocyte, neutrophil, RDW-CV, AST, NLR, SIRI, APRI, RPR, and DNLR levels increased markedly. These diverse post-ACS inflammatory responses highlight that the immunomodulatory effects of ACS in preeclamptic pregnancies are complex and not yet fully understood, underscoring the need for further well-designed prospective studies to elucidate their clinical implications.

A meta-analysis demonstrated that elevated MPV values were associated with PE.^[37]^ Our study found that the ΔMPV value was positively correlated with gestational age at delivery and birth weight. A recent study examining neonatal outcomes in relation to MPV found that, in line with our findings, the need for prolonged oxygen support and intubation increased significantly in infants exposed to MPV levels above 10 fL.^[38]^

In a study of a large cohort, the NLR was found to be significantly higher and the PLR significantly lower in preeclamptic women than in non-preeclamptic women just before delivery.^[39]^ The high NLR and low PLR values in the PE group are also consistent with our study. The difference in our study is that we found a positive correlation between ΔNLR following ACS, gestational age at delivery and birth weight.

### 7.4. The strengths and limitations

Strengths of this study include the examination of dynamic changes in inflammatory markers following ACS therapy in pregnant women with PE, which has not been previously investigated. Additionally, by examining the relationship between these hematologic alterations and perinatal outcomes, the study identified that ΔAPRI and ΔMPV may have predictive value for NICU admission. The large sample size and comprehensive analysis of a variety of inflammatory markers and indices add to the strength of the study.

Limitations of this study include its retrospective design, which may introduce bias and limit the ability to establish causality. The study also focused on a specific population of pregnant women with PE who received ACS therapy, which may limit the generalizability of the findings to a broader population. Although potential confounding factors such as maternal autoimmune or chronic inflammatory diseases, active infections, diabetes, and those receiving a single rescue dose of betamethasone were excluded, other unmeasured clinical or therapeutic variables might still have influenced the outcomes. Further research is needed to validate the findings of this study and explore the potential mechanisms underlying the observed changes in inflammatory markers following ACS therapy.

## 8. Conclusion

This study suggests that ACS therapy may be associated with dynamic alterations in maternal inflammatory markers among women with PE, which could have potential implications for perinatal outcomes. The observed variations in post-ACS indices, particularly ΔMPV and ΔAPRI, appeared to show an association with the likelihood of NICU admission, indicating a possible predictive value. However, given the retrospective design, these findings should be interpreted with caution and regarded as exploratory. Further prospective studies are needed to confirm these associations and to elucidate the underlying mechanisms through which ACS may influence inflammatory responses in PE. Understanding these interactions could contribute to more refined risk assessment and management strategies in this complex clinical setting.

## Author contributions

**Conceptualization:** Sebnem Karagun, Ahmet Zeki Nessar, Ayhan Coşkun.

**Data curation:** Sebnem Karagun, Ahmet Zeki Nessar, Sefanur Gamze Karaca, Hamza Yildiz.

**Formal analysis:** Sebnem Karagun, Ahmet Zeki Nessar, Hamza Yildiz.

**Funding acquisition:** Sebnem Karagun, Yusuf Dal.

**Investigation:** Sebnem Karagun, Yusuf Dal, Hamza Yildiz.

**Methodology:** Sebnem Karagun, Yusuf Dal, Hamza Yildiz.

**Project administration:** Sebnem Karagun, Sefanur Gamze Karaca, Mürşide Çevikoğlu Killi, Hamza Yildiz, Ayhan Coşkun.

**Resources:** Sebnem Karagun, Sefanur Gamze Karaca, Mürşide Çevikoğlu Killi.

**Software:** Sebnem Karagun, Sefanur Gamze Karaca, Mürşide Çevikoğlu Killi.

**Supervision:** Sebnem Karagun, Yusuf Dal, Sefanur Gamze Karaca, Mürşide Çevikoğlu Killi, Ayhan Coşkun.

**Validation:** Sebnem Karagun, Sefanur Gamze Karaca, Mürşide Çevikoğlu Killi, Ayhan Coşkun.

**Visualization:** Sebnem Karagun, Mürşide Çevikoğlu Killi.

**Writing – original draft:** Sebnem Karagun.

**Writing – review & editing:** Sebnem Karagun, Ayhan Coşkun.
